# Meta-signature of human endometrial receptivity: a meta-analysis and validation study of transcriptomic biomarkers

**DOI:** 10.1038/s41598-017-10098-3

**Published:** 2017-08-30

**Authors:** Signe Altmäe, Mariann Koel, Urmo Võsa, Priit Adler, Marina Suhorutšenko, Triin Laisk-Podar, Viktorija Kukushkina, Merli Saare, Agne Velthut-Meikas, Kaarel Krjutškov, Lusine Aghajanova, Parameswaran G. Lalitkumar, Kristina Gemzell-Danielsson, Linda Giudice, Carlos Simón, Andres Salumets

**Affiliations:** 1 Department of Women’s and Children’s Health, Division of Obstetrics and Gynecology, Karolinska Institutet, and Karolinska University Hospital, 17176 Stockholm, Sweden; 2 Competence Centre on Health Technologies, 50410 Tartu, Estonia; 3 Department of Biochemistry and Molecular Biology, Faculty of Sciences, University of Granada, 18016 Granada, Spain; 4 Department of Biosciences and Nutrition, and Center for Innovative Medicine, Karolinska Institutet, 14183 Huddinge, Sweden; 5 Department of Cell Biology, Institute of Molecular and Cell Biology, University of Tartu, 51010 Tartu, Estonia; 6 Estonian Genome Center, University of Tartu, 51010 Tartu, Estonia; 7 Institute of Computer Science, University of Tartu, Tartu, 50409 Estonia; 8 Department of Obstetrics and Gynaecology, Institute of Clinical Medicine, University of Tartu, 51014 Tartu, Estonia; 9 Department of Obstetrics, Gynecology, and Reproductive Sciences, University of California San Francisco, San Francisco, 94143-0132 CA USA; 10 Department of Obstetrics and Gynaecology, Valencia University & INCLIVA, Igenomix & Fundación IVI, 46021 Valencia, Spain; 11 Department of Obstetrics and Gynecology, University of Helsinki and Helsinki University Hospital, Helsinki, FI-00029 HUS, Finland

## Abstract

Previous transcriptome studies of the human endometrium have revealed hundreds of simultaneously up- and down-regulated genes that are involved in endometrial receptivity. However, the overlap between the studies is relatively small, and we are still searching for potential diagnostic biomarkers. Here we perform a meta-analysis of endometrial-receptivity associated genes on 164 endometrial samples (76 from ‘pre-receptive’ and 88 from mid-secretory, ‘receptive’ phase endometria) using a robust rank aggregation (RRA) method, followed by enrichment analysis, and regulatory microRNA prediction. We identify a meta-signature of endometrial receptivity involving 57 mRNA genes as putative receptivity markers, where 39 of these we confirm experimentally using RNA-sequencing method in two separate datasets. The meta-signature genes highlight the importance of immune responses, the complement cascade pathway and the involvement of exosomes in mid-secretory endometrial functions. Bioinformatic prediction identifies 348 microRNAs that could regulate 30 endometrial-receptivity associated genes, and we confirm experimentally the decreased expression of 19 microRNAs with 11 corresponding up-regulated meta-signature genes in our validation experiments. The 57 identified meta-signature genes and involved pathways, together with their regulatory microRNAs could serve as promising and sought-after biomarkers of endometrial receptivity, fertility and infertility.

## Introduction

The period of endometrial receptivity, also known as the window of implantation (WOI), is the limited time (one to two days) when luminal epithelium is favourable for embryo adhesion as the first step of implantation^1^. Successful embryo implantation depends on synchronization of a viable embryo and receptive endometrium. In fact, inadequate uterine receptivity has been estimated to contribute to one third of implantation failures, whereas the embryo itself is responsible for two thirds of them^2, 3^. In assisted reproductive technologies where good-quality embryos are transferred as a standard of care, implantation failure remains an unsolved obstacle^4–6^. In patients with recurrent implantation failure (RIF) temporal displacement of the WOI has been described in one out of four patients^7^, thus suggesting the possibility of these women suffering RIF of endometrial origin. Further, impaired decidualization of endometrial stromal cells that predisposes to late implantation may negate endometrial ‘embryo quality control’ and cause early pregnancy failure^8–10^. Hence, better understanding of endometrial receptivity and the importance of the mechanisms involved in mid-secretory endometrial functions is warranted.

From the first histological dating methods^11, 12^ to the new ‘omics’ technologies, extensive efforts have been made to understand and characterise receptive endometrium. Traditional endometrial dating criteria, like tissue histology, are obsolete, since their accuracy, reproducibility and functional relevance have been questioned in various randomised studies^13, 14^. This has encouraged further investigation and application of new technologies to diagnose endometrial receptivity objectively, since reliable diagnostic markers are still lacking and the molecular mechanisms remain largely unclear^15–17^.

With the ‘omics’ revolution, the quest for the transcriptomic signature of human endometrial receptivity has revealed hundreds of simultaneously up- and down-regulated genes implicated in the phenomenon (reviewed in ref. 18). While any given study yields a number of genes, the overlap between different studies is relatively small. The perceived limitations of this technology have been well defined and lie in differences in experimental design, timing and conditions of endometrial sampling, selection criteria regarding patients, transcriptome array/sequencing platforms and genome annotation versions used, pipelines for data processing and a lack of consistent standards for data presentation^19–23^.

To overcome the aforementioned limitations in endometrial transcriptome analyses, we applied a recently published robust rank aggregation (RRA) method^24^, followed by enrichment analysis, to identify a meta-signature or consensus signature of highly putative biomarkers of endometrial receptivity. Additionally, we set up to analyse possible microRNAs that could influence the endometrial receptivity-associated genes/mRNAs. Further, we aimed to experimentally validate the meta-signature mRNA genes and their regulatory miRNAs in two independent sample sets.

## Results

### Identification of relevant studies

The search process and results of the systematic literature review are presented in detail in Supplementary Figure 1. Eventually, out of 57 eligible publications, 14 remained suitable for qualitative analysis. Five eligible studies^25–29^ were not included in the final analysis, since the data on lists of differentially expressed genes were not available publicly nor in response to requests to the authors. A detailed description of the studies included in the final analysis is presented in Table 1. Our pooled dataset obtained from the nine remaining studies covered 76 ‘pre-receptive’-phase (28 biopsy samples from the proliferative phase and 48 from the early secretory phase) and 88 mid-secretory, ‘receptive’ phase endometrial samples.

**Table 1:** Characteristics of the analysed datasets. ES indicates early secretory phase, MS – mid-secretory phase, cd – cycle day, LH – luteinizing hormone, FC – fold change, N/S – not specified, * – samples pooled for microarray analysis, ** – ERA test training that was performed on 68 additional endometrial samples.

First author and reference	Participants	Region	Biopsy obtained	Cycle dating	First sample (day, n)	Second sample (day, n)	Array/sequencing platform	FC (cut-off)	Up-regulated transcripts (n)	Down-regulated transcripts (n)
Mid-secretory *vs*. proliferative										
Kao^85^	Normally cycling women	North America	Pipelle catheter	urinary LH	cd 8–10, n = 4	LH+8-10, n = 7	Affymetrix Hu95A	≥2.0	156	377
Borthwick^86^	Regular cycles, normal pelvis	Europe	N/S	urinary LH	cd 9-11, n = 5	LH+6-8, n = 5	Affymetrix Hu95A-E*	≥2.0	90	46
Altmäe^37^	Healthy fertile volunteers	Europe	Pipelle catheter	urinary LH	cd 7, n = 4	LH+7, n = 4	Affymetrix HG-U133 plus 2.0	p < 0.05	920	1257
Mid-secretory *vs*. early secretory										
Carson^87^	Fertile volunteers	North America	Pipelle catheter	urinary LH	LH+2-4, n = 3	LH+7-9, n = 3	Affymetrix Hu95A*	≥2.0	323	370
Riesewijk^88^	Normally cycling women	Europe	Pipelle catheter	urinary LH	LH+2, n = 5	LH+7, n = 5	Affymetrix Hu95A	≥3.0	153	58
Mirkin^89^	Healthy fertile oocyte donors	North America	Pipelle catheter	urinary LH	LH+3, n = 3	LH+8, n = 5	Affymetrix HG-U95Av2	≥2.0	49	58
Talbi^33^	Normally cycling women	North America	Pipelle catheter	Noyes	ES, n = 3	MS, n = 8	Affymetrix HG-U133 plus 2.0	≥1.5	1415	1463
Diaz-Gimeno^30^	Healthy fertile oocyte donors	Europe	Pipelle catheter	urinary LH	LH+1, n = 5; LH+3, n = 5; LH+LH+5, n = 5;**cd 8-12, n = 15; **LH+1–+5, n = 13; **LH+7, n = 40	LH+7, n = 5	Agilent Whole Human Genome Oligo Microarray	≥3.0	143	95
Hu^38^	Normally cycling women	Asia	Pipelle catheter	urinary LH	LH+2, n = 6	LH+7, n = 6	RNA-seq Illumina Genome Analyzer IIx	p < 0.001, FC > 2	1099	1273

### Meta-signature of endometrial receptivity-associated genes

Using robust rank aggregation analysis, we identified a statistically significant meta-signature of 52 up-regulated and five down-regulated genes in mid-secretory *vs*. ‘pre-receptive’ endometrium (see Table 2). The up-regulated transcripts with the highest scores in receptive-phase endometrium were *PAEP*, *SPP1*, *GPX3*, *MAOA* and *GADD45A*. The five down-regulated transcripts identified as receptivity-associated genes were *SFRP4*, *EDN3*, *OLFM1*, *CRABP2* and *MMP7*.

**Table 2:** List of genes identified as specific biomarkers of mid-secretory endometrium when assessed in comparative transcriptome analyses with proliferative and early secretory endometrium in nine datasets. 52 genes are up-regulated in mid-secretory endometrium, while five are down-regulated (↓).

**ENTREZ ID**	**HUGO Symbol**	**HUGO Name**	VAL*	VAL**	**RRA score**	**Adjusted P**
5047	***PAEP*** ^b,c,d,e^	Progestagen-associated endometrial protein		90	7.68E-18	2.99E-13
6696	***SPP1*** ^b,d,e^	Secreted phosphoprotein 1 (osteopontin)	30, 32, 87, 89, 91	92	2.06E-15	8.04E-11
2878	***GPX3*** ^b,c,d,e^	Glutathione peroxidase 3	30, 86, 88, 91		1.89E-14	7.40E-10
4128	***MAOA*** ^a,b,d,e^	Monoamine oxidase A	17	93 +	2.32E-13	9.04E-09
1647	*GADD45A* ^b,c,d,e^	Growth arrest and DNA-damage-inducible, alpha			2.73E-13	1.06E-08
22943	***DKK1*** ^a,b,d,e^	Dickkopf WNT signalling pathway inhibitor 1	17, 33, 85	94	2.80E-13	1.09E-08
1364	***CLDN4*** ^a,b,d,e^	Claudin 4	87, 88	87, 95	9.14E-13	3.57E-08
722	***C4BPA*** ^b,d,e^	Complement component 4 binding protein, alpha	91	56	1.60E-12	6.23E-08
3600	*IL15* ^a,b,d,e^	Interleukin 15		96	3.36E-12	1.31E-07
1604	*CD55* ^a,b,d,e^	CD55 molecule, decay accelerating factor for complement	33	97	5.47E-12	2.14E-07
3400	*ID4* ^b^	Inhibitor of DNA binding 4, dominant negative helix-loop-helix protein			6.43E-12	2.51E-07
10578	*GNLY* ^b,d^	Granulysin		98+	1.78E-11	6.96E-07
1356	***CP*** ^a,b,d^	Ceruloplasmin			2.81E-11	1.10E-06
6505	*SLC1A1* ^a,d,e^	Solute carrier family 1, member 1	88		5.27E-11	2.06E-06
1803	***DPP4*** ^b,c,d,e^	Dipeptidyl-peptidase 4	32	99, 100	7.44E-11	2.90E-06
6947	***TCN1*** ^d,e^	Transcobalamin I			1.54E-10	6.03E-06
1675	*CFD* ^a,b,d^	Complement factor D			2.56E-10	9.99E-06
307	***ANXA4*** ^a,b,d,e^	Annexin A4	17, 89, 91	101	1.50E-09	5.85E-05
1942	***EFNA1*** ^d^	Ephrin-A1		102	1.55E-09	6.06E-05
2634	***GBP2*** ^a,b,d^	Guanylate binding protein 2, interferon-inducible			1.63E-09	6.38E-05
347	***APOD*** ^b,d,e^	Apolipoprotein D	85, 91	103	3.05E-09	1.19E-04
604	*BCL6* ^a,d^	B-cell CLL/lymphoma 6		104	4.42E-09	1.72E-04
1052	*CEBPD* ^d^	CCAAT/enhancer binding protein, delta		105	5.09E-09	1.99E-04
36	*ACADSB* ^e^	Acyl-CoA dehydrogenase, short/branched chain			6.16E-09	2.40E-04
11067	***C10orf10*** ^a,d^	Chromosome 10 open reading frame 10			7.24E-09	2.83E-04
8714	***ABCC3*** ^a,d,e^	ATP-binding cassette, sub-family C, member 3			7.53E-09	2.94E-04
4495	***MT1G*** ^b,d,e^	Metallothionein 1G	30, 38, 86		8.16E-09	3.19E-04
384	***ARG2*** ^a,d^	Arginase 2		106	8.52E-09	3.33E-04
1311	*COMP* ^b,d,e^	Cartilage oligomeric matrix protein		32	9.02E-09	3.52E-04
50486	***G0S2*** ^b,e^	G0/G1 switch 2			1.10E-08	4.31E-04
7103	***TSPAN8*** ^a,d,e^	Tetraspanin 8			1.22E-08	4.76E-04
1672	***DEFB1*** ^a,d^	Defensin, beta 1		107	1.43E-08	5.57E-04
4217	*MAP3K5* ^b,d,e^	Mitogen-activated protein kinase kinase kinase 5			1.54E-08	6.00E-04
1910	***EDNRB*** ^c,d,e^	Endothelin receptor type B		108+	2.34E-08	9.15E-04
158471	***PRUNE2*** ^b^	Prune homolog 2			3.48E-08	1.36E-03
6286	*S100P* ^a,b,d,e^	S100 calcium binding protein P	17, 28	25, 28	4.01E-08	1.56E-03
3484	*IGFBP1* ^e^	Insulin-like growth factor binding protein 1	33, 85	109	4.92E-08	1.92E-03
11056	***DDX52***	DEAD (Asp-Glu-Ala-Asp) box polypeptide 52			5.97E-08	2.33E-03
710	***SERPING1*** ^a,b,d^	Serpin peptidase inhibitor, clade G, member 1			7.05E-08	2.75E-03
84159	***ARID5B*** ^d,e^	AT rich interactive domain 5B			8.69E-08	3.40E-03
3914	***LAMB3*** ^b,d,e^	Laminin, beta 3	26		1.45E-07	5.66E-03
316	***AOX1*** ^a,d,e^	Aldehyde oxidase 1	91		2.09E-07	8.17E-03
3620	***IDO1***	Indoleamine 2,3-dioxygenase 1		110	2.67E-07	1.04E-02
302	***ANXA2*** ^b,e^	Annexin A2		111	3.26E-07	1.27E-02
3026	***HABP2*** ^c,d,e^	Hyaluronan binding protein 2		112	3.26E-07	1.27E-02
715	***C1R*** ^b,e^	Complement component 1, r subcomponent			3.26E-07	1.27E-02
360	*AQP3* ^e^	Aquaporin 3		113	4.27E-07	1.66E-02
6990	***DYNLT3*** ^a^	Dynein, light chain, Tctex-type 3			5.07E-07	1.98E-02
4496	*MT1H* ^d,e^	Metallothionein 1 H		114	8.16E-07	3.18E-02
4837	***NNMT*** ^d^	Nicotinamide N-methyltransferase	91		8.74E-07	3.41E-02
10397	***NDRG1*** ^d^	N-myc downstream regulated 1		115	1.01E-06	3.95E-02
2028	*ENPEP*	Glutamyl aminopeptidase			1.11E-06	4.32E-02
6424	***SFRP4***↓^b^	Secreted frizzled-related protein 4	17		5.95E-11	2.32E-06
1908	***EDN3***↓^b^	Endothelin 3		108+	3.06E-10	1.20E-05
10439	***OLFM1***↓^a,b^	Olfactomedin 1		116	1.65E-09	6.46E-05
1382	***CRABP2***↓^a,b^	Cellular retinoic acid binding protein 2		56	1.97E-08	7.68E-04
4316	*MMP7*↓^a,b^	Matrix metallopeptidase 7			4.07E-07	1.59E-02

Genes validated in our independent sample sets of endometrial samples at LH+2 *vs*. LH+8 from healthy fertile women analysed with RNA-seq and cell type-specific RNA-seq methods are highlighted in **bold**. Genes present in the ERA diagnostic tool are underlined. Genes also identified in previous data-mining/review studies are indicated in super-scripts: a^17^, b^31^, c^19^, d^29^, and e^32^.

VAL* indicates mRNA validation experiments in previous transcriptomic studies on mid-secretory endometrium using real-time PCR, Northern blot or *in situ* hybridisation analyses.

VAL** indicates protein validation analyses in mid-secretory endometrium. ^+^ stands for validation in other species (mouse, bovine or rhesus monkey).

### Enrichment analyses

**Figure 1:**
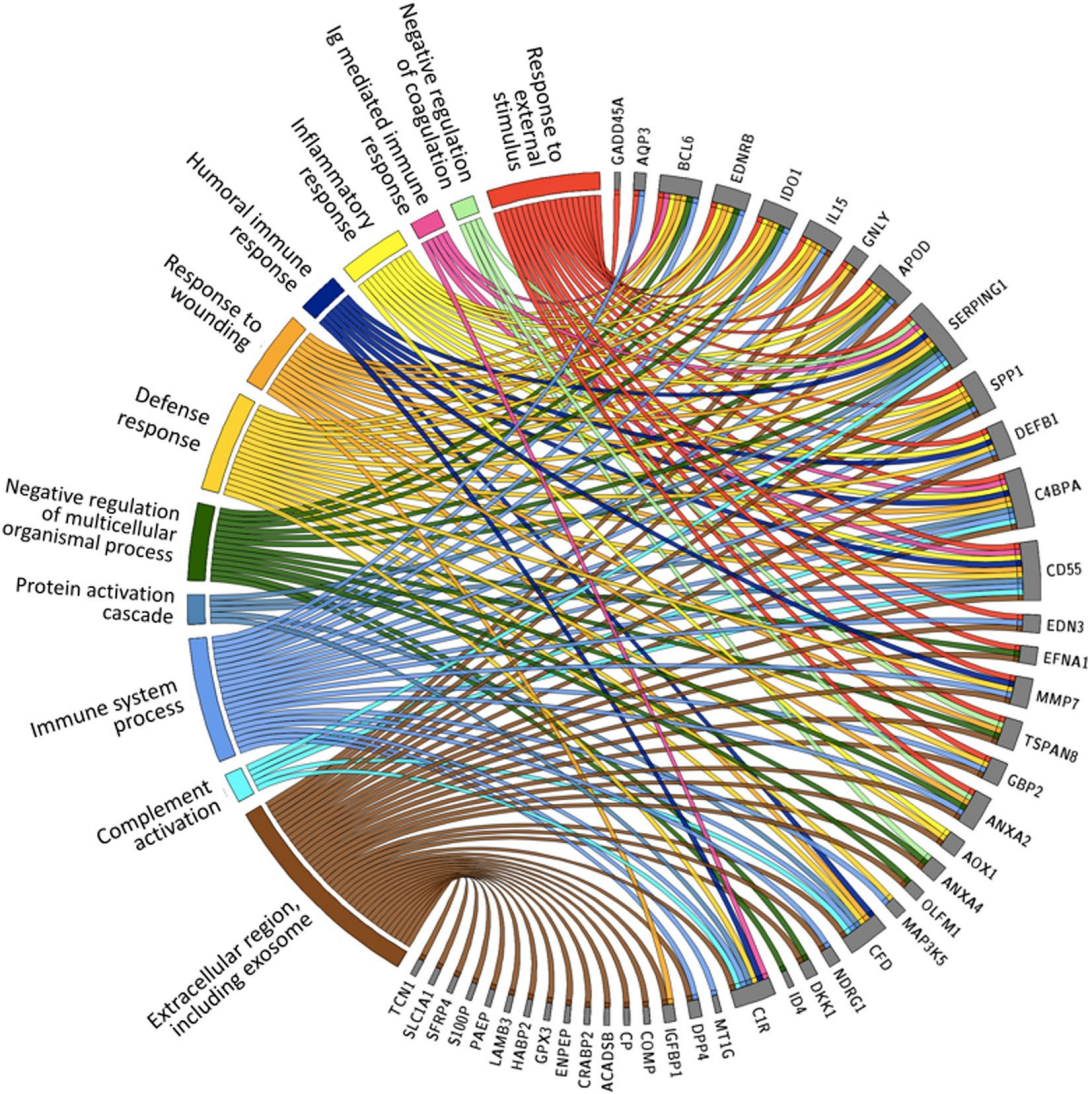
Gene ontology (GO) processes and the pathways most strongly enriched among endometrial receptivity-associated genes. Genes are presented on the right side on the circle and the correlating GO processes, cellular compartments and pathways are on the left side.

**Figure 2:**
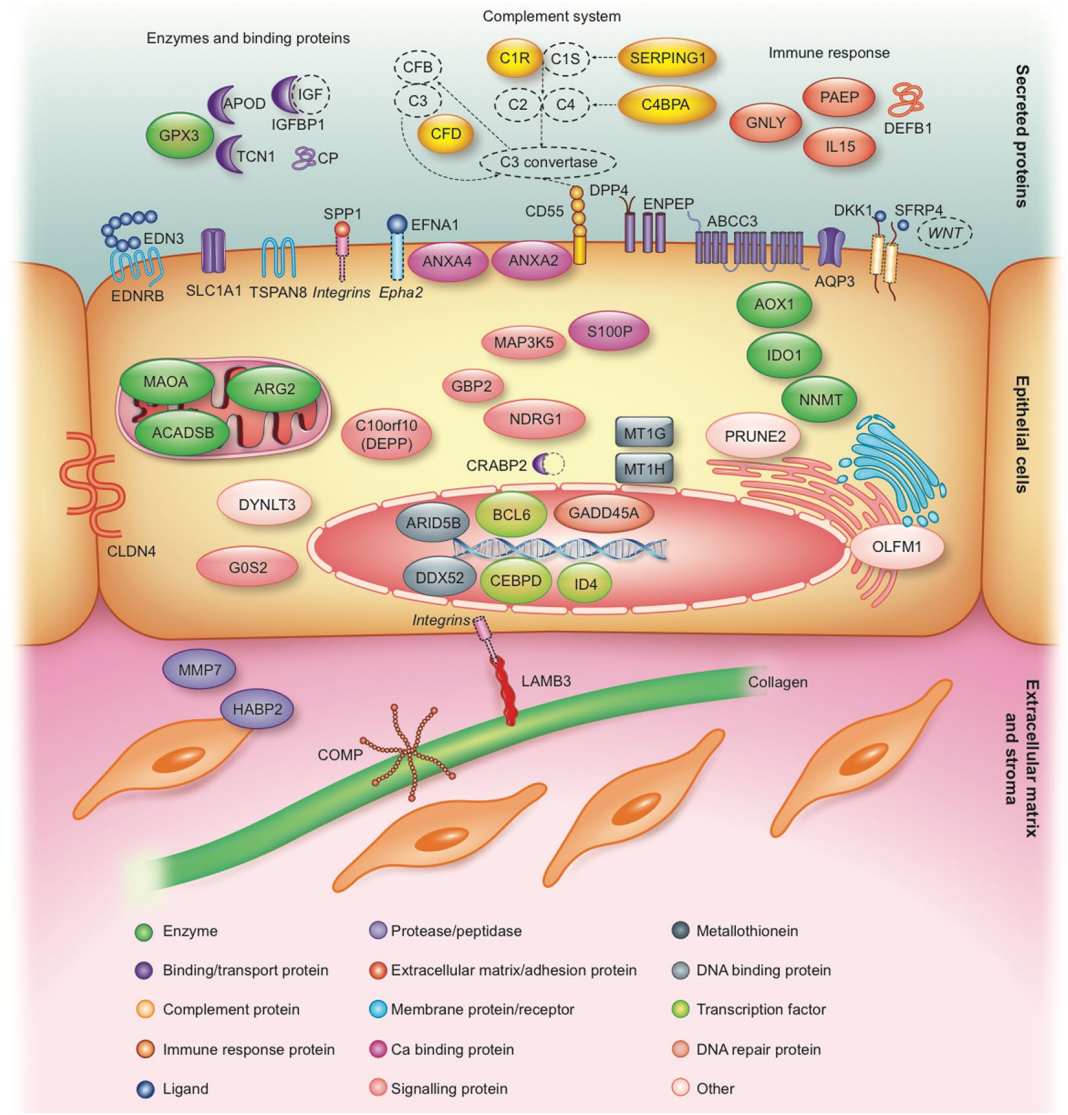
Schematic overview of the 57 meta-signature genes, their literature-based localisation and involvement in the mid-secretory phase endometrium. Different membrane-associated proteins (ABCC3, ANXA2, ANXA4, AQP3, CD55, DKK1, DPP4, EDN3, EDNRB, EFNA1, ENPEP, SFRP4, SLC1A1, SPP1, TSPAN8), epithelial cell tight junction protein (CLDN4), secreted enzymes and binding proteins (APOD, CP, GPX3, IGFBP1, TCN1), secreted immune response proteins (DEFB1, GLNY, IL15, PAEP), extracellular matrix-associated proteins (COMP, HABP2, LAMB3, MMP7), different enzymes (ACADSB, AOX1, ARG2, IDO1, MAOA, NNMT), signalling proteins (C10orf10, GBP2, G0S2, MAP3K5, NDRG1), metallothioneins (MT1G, MT1H), DNA binding and repair proteins (ARID5B, DDX52, GADD45A), transcription factors (BCL6, CEBPD, ID4), and other intracellular proteins (CRABP2, DYNLT3, OLFM1, PRUNE2, S100P) are indicated. Additionally, the enriched KEGG pathway of complement cascade with the identified genes *C1R*, *SERPING1*, *CD55*, *C4BPA* and *CFD* is highlighted. (Figure created by Elsevier Illustration Service).

**Figure 3:**
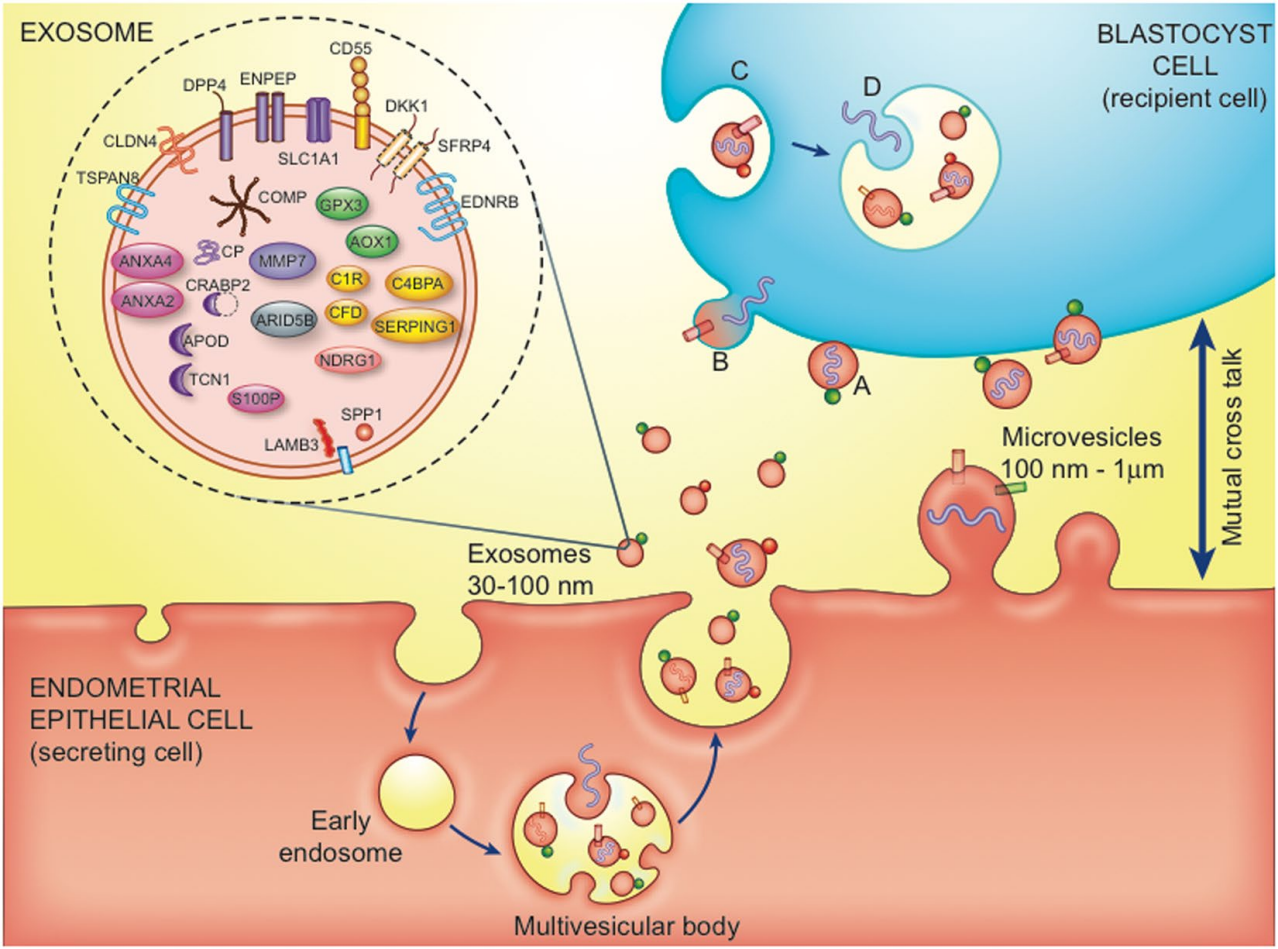
Extracellular vesicles (EVs) – exosomes and microvesicles, in embryo-endometrium cross-talk. In the exosomes the meta-signature genes are highlighted (based on ExoCarta database). Exosomes (30–100 nm) are generated from inward budding of the endosomal membrane, resulting in formation of a multivesicular body. Microvesicles (100 nm–1 μm) are produced by direct budding of the plasma membrane. Membrane-associated (bubbles) and transmembrane proteins (cylinders), and nucleic acids (DNA, RNA, curved symbols) are selectively incorporated into the EVs. EVs may dock on the plasma membrane of a target cell (**A**), fuse directly with the plasma membrane (**B**), or be endocytosed (**C**). Endocytosed vesicles may subsequently fuse with the delimiting membrane of an endocytic compartment (**D**). Both (**B** and **D**) pathways result in the delivery of proteins and nucleic acids into the membrane or cytosol of the target cell. (Figure adapted with permission from^62, 84^, created by Elsevier Illustration Service).

We used up-to-date enrichment analysis software (g:Profiler) for analysis of biological processes and pathways connected to the meta-signature of mid-secretory endometrium. A significant proportion of the genes were involved in biological processes such as responses to external stimuli, responses to wounding, inflammatory responses, negative regulation of coagulation, humoral immune responses, and immunoglobulin-mediated immune responses, among others. Fig. 1 and Supplementary Table 1 show the connections of the 57 endometrial receptivity genes with their respective Gene Ontology biological processes. The only significantly enriched pathway related to the meta-signature genes was a KEGG pathway of complement and coagulation cascades, where the identified genes were connected to the complement cascade part (p = 0.00112) (see Fig. 2). A significant number of the genes were also connected with the extracellular region and exosomes. In order to confirm the involvement of exosomes, we searched for the presence of the meta-signature genes in human exosomes based on the exosome database, ExoCarta (exocarta.org). Fisher’s Exact Test was performed to analyse if meta-signature genes were over represented in the exosome database. All the human protein coding genes were downloading from ENSEMBL v75 database (version February 2014) and mRNAs or proteins from Exocarta database (exocarta.org). Altogether, meta-signature genes had 2.13 times higher probability to be in the exosomes than the rest of the protein-coding genes in the human genome (Fisher’s exact test, two-sided p = 0.0059). The 28 identified proteins from the meta-signature gene list that have been shown to be in exosomes are presented in Fig. 3 that illustrates the involvement of extracellular vesicles (exosomes and microvesicles) in embryo implantation process.

### Validation of meta-signature genes in two independent sample sets

**Figure 4:**
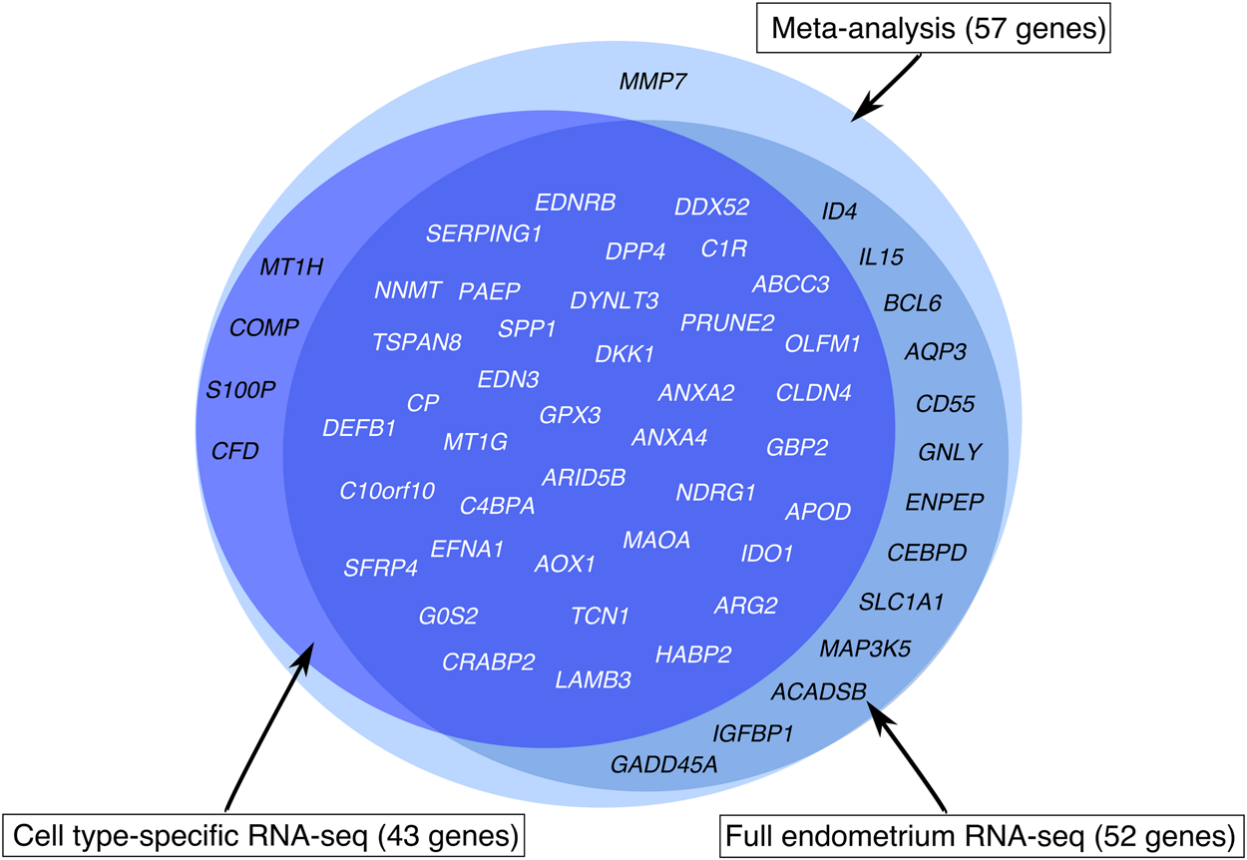
Validation of the meta-signature genes in two independent sample sets. RNA-seq analysis of endometrial tissue samples confirmed differential expression of 52 (91.2%) meta-signature genes in the mid-secretory phase endometrium *vs*. early secretory phase endometrium. Cell type-specific RNA-seq analysis of endometrial epithelial and stromal cells confirmed differential expression of 43 (75.4%) meta-signature genes in those cell populations in the mid-secretory endometrium *vs*. early secretory endometrium. In total, 39 (68.4%) meta-signature genes (typed in white colour) were identified in validation experiments on two different sample sets, where 35 genes were up-regulated and 4 genes (*CRABP2*, *EDN3*, *OLFM1*, *SFRP4*) down-regulated in the mid-secretory phase endometrium.

Meta-analysis identified 57 genes differentially expressed between the ‘pre-receptive’ and mid-secretory endometrium, where 52 genes were up- and five were down-regulated at WOI. Our RNA-sequencing (RNA-seq) analysis on 20 independent endometrial biopsy samples from fertile women confirmed the differential expression of 52 meta-signature genes (all of them with fold change of ≥3) – 48 of these genes were likewise up-regulated and four were down-regulated (*CRABP2*, *EDN3*, *OLFM1*, *SFRP4*) in the mid-secretory endometria (Fig. 4). *MMP7* and *CFD* were not differentially expressed in our RNA-seq analysis of LH+8 *vs*. LH+2 phase endometria. Three genes, *COMP*, *MT1H*, *S100P*, did not pass the initial filtering of RNA-seq data (counts per million, CPM > 2 in at least 15 samples), which might be due to their low expression levels. The filtering was applied to rule out transcripts with very low or inconsistent expression levels across individuals.

**Figure 5:**
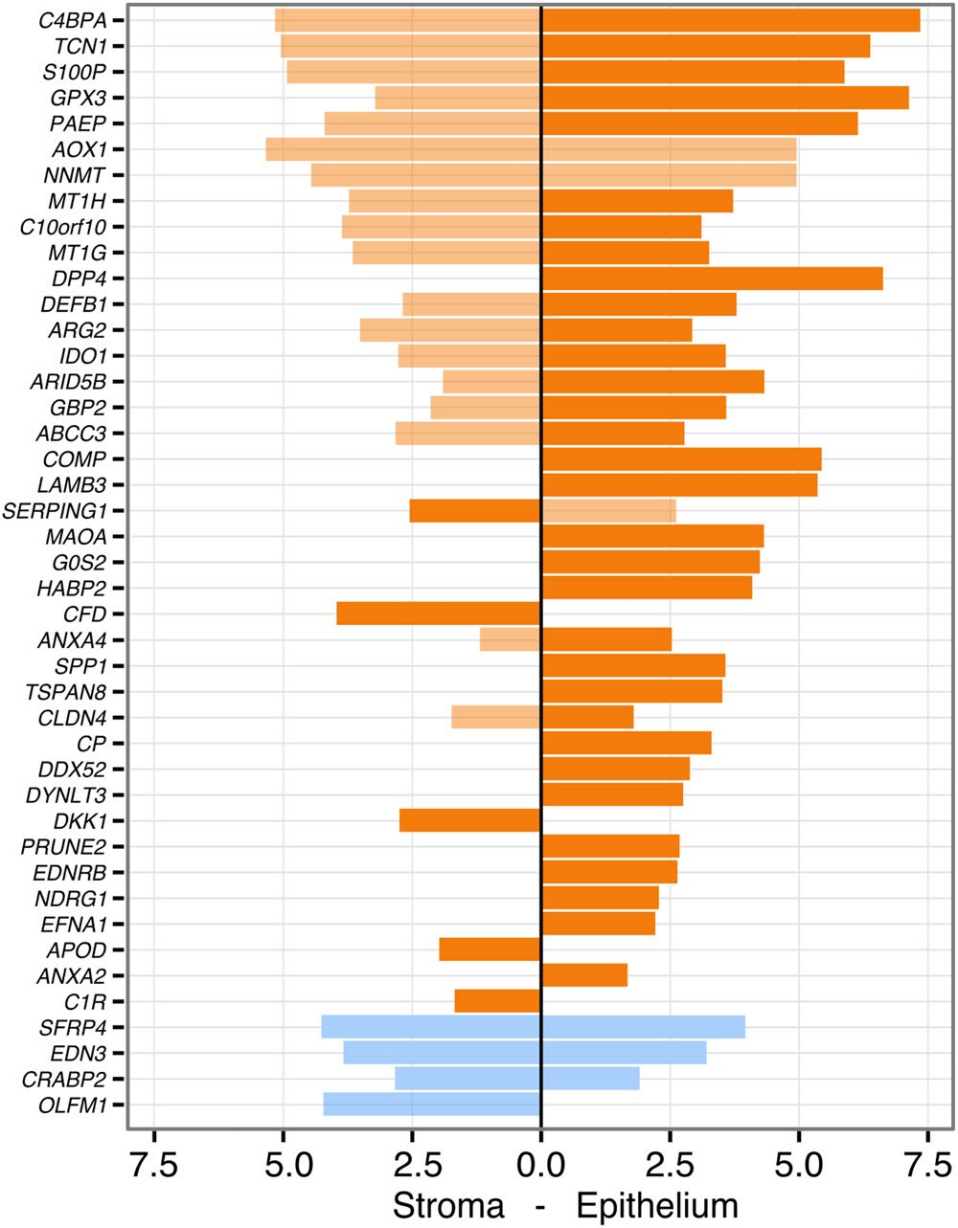
Validation of the meta-signature genes on cell type-specific RNA-seq data. Significantly up-regulated (orange) and down-regulated (blue) genes in FACS-sorted stromal and epithelial cells. The x-scale represents log_2_(FC) between LH+8 *vs*. LH+2 comparisons in stromal and epithelial cells. When comparing the gene expression values between epithelial *vs*. stromal cells in the mid-secretory phase endometrium (LH+8), most genes were more up-regulated in the epithelial cells (higher expression highlighted as darker orange). All reported results are significant at FDR < 0.05.

Next, we investigated the expression of the 57 meta-signature genes in FACS (fluorescence-activated cell sorting)-sorted endometrial epithelial and stromal cells from two time points in the menstrual cycle, early secretory *vs*. mid-secretory phase, from 16 fertile women. Thirty-nine of those genes were significantly up-regulated and four were down-regulated (*CRABP2*, *EDN3*, *OLFM1*, *SFRP4*) in the receptive phase in those cell populations (all of them with fold change of ≥2) (Fig. 4; Supplementary Figure 2). Although most of the genes were up-regulated in both cell types, it is notable that the expression of *ANXA2*, *COMP*, *CP*, *DDX52*, *DPP4*, *DYNLT3*, *EDNRB*, *EFNA1*, *G0S2*, *HABP2*, *LAMB3*, *MAOA*, *NDRG1*, *PRUNE2*, *SPP1*, and *TSPAN8* was epithelium-specific (Fig. 5), while none of the genes was down-regulated in the epithelial cells only. The stroma-specific up-regulated genes were *APOD*, *CFD*, *C1R* and *DKK1*, and down-regulated gene was *OLFM1* (Fig. 5). It is noteworthy that although most of the genes were up-regulated in both cell types, the expression of these genes was still higher in the epithelial cells.

Further validation of these confirmed meta-signature genes was carried out with real-time PCR. Up-regulation of *DDX52*, *DYNLT3*, *C1R* and *APOD* expression levels in the receptive phase endometrial samples was confirmed (Supplementary Figure 3). Furthermore, the cell-specific up-regulated *DDX52* and *DYNLT3* expression was confirmed in FACS-sorted epithelial cells, and the stromal cell-specific *C1R* and *APOD* up-regulation was confirmed in the FACS-sorted stromal cells (Supplementary Figure 3).

In conclusion, the validation of the 57 meta-analysis consensus genes of the receptive phase endometrium among the two independent sets of endometrial tissue samples and cell-populations analysed confirmed the differential expression of 39 genes, with 35 up- and 4 down-regulated expression during WOI (Fig. 4).

### *In silico* analysis of potential microRNAs regulating meta-signature genes

To evaluate the potential regulation of the 57 meta-signature genes, we predicted their putative regulatory-microRNAs using three different *in silico* target prediction algorithms. DIANA microT-CDS predicted 1,355 microRNAs with 12,627 potential binding sites, TargetScan 7.0 predicted 2,521 microRNAs with 32,560 potential binding sites, and miRanda predicted 2,568 microRNAs with 42,413 potential binding sites. The overlap between all three algorithms resulted in 818 microRNAs and 1,403 potential unique binding sites for 43 meta-signature genes (Supplementary Table 2).

To add an additional filter to the bioinformatic predictions, we overlaid those with experimentally determined Argonaute binding sites (microRNAs regulate gene expression by guiding Argonaute proteins to specific target mRNA sequences), mined from publicly available AGO-CLIP datasets. Out of 1,403 intersected potential binding sites, 395 showed overlap with experimentally determined Argonaute binding site in human cell lines, filtering down to the most probable microRNA and mRNA interactions. These 395 sites included interactions between 30 genes from our original meta-signature gene list and 348 microRNAs (Supplementary Table 3).

### Validation analysis of predicted microRNAs and target mRNAs in independent sample set

**Figure 6:**
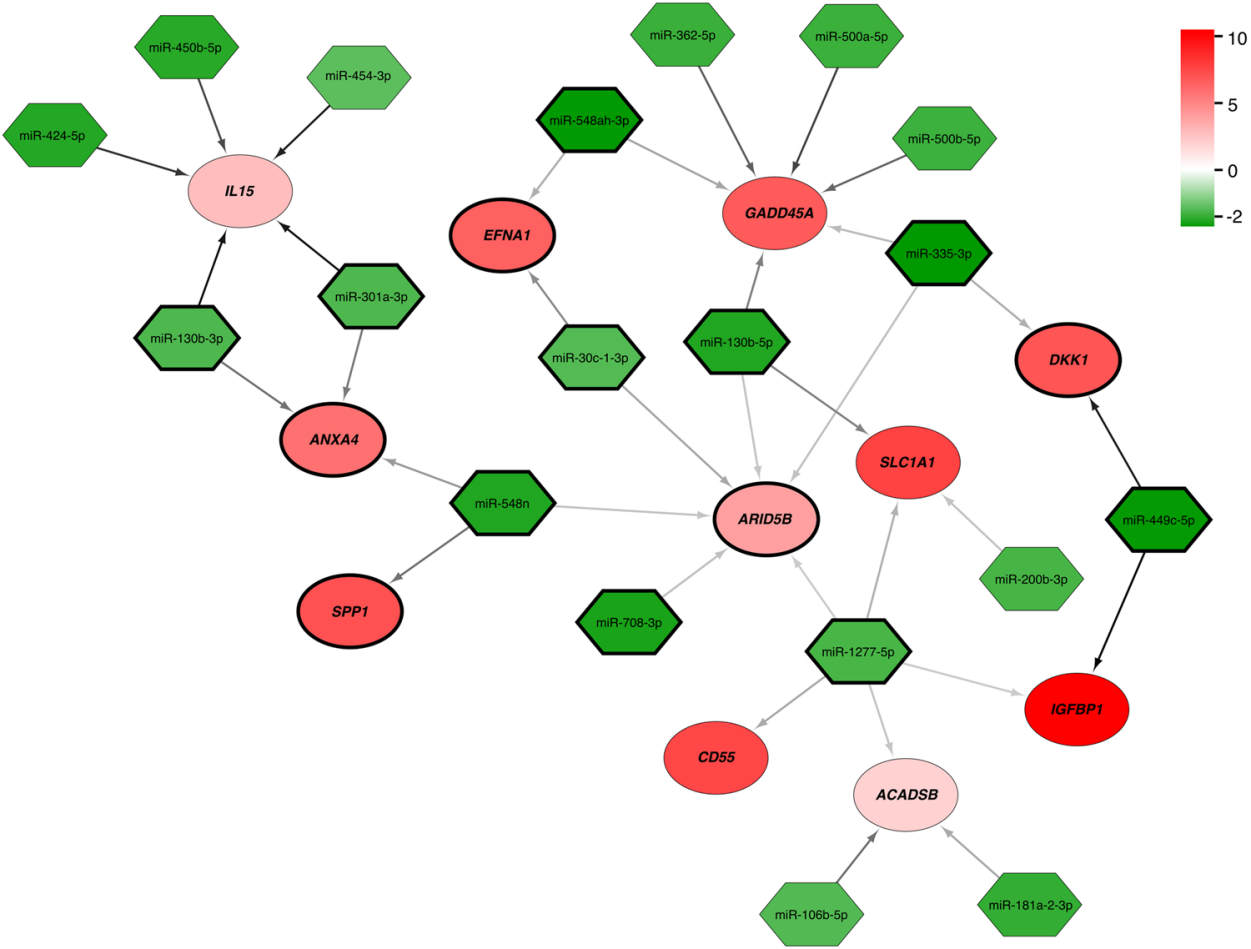
*In silico* predicted interactions between significantly up-regulated mRNAs (red) and down-regulated microRNAs (green) in LH+8 *vs*. LH+2 endometrium. The colour intensity indicates the strength of up- or down-regulation (FDR < 0.05). The colour of the arrows between the microRNA and mRNA represents TargetScan context++ score (see Supplementary Table 2 for scores), where darker arrow shows more probable interaction. The number of arrows between microRNA and mRNA indicates different microRNA binding sites within the same transcript. Meta-signature genes that were confirmed in both independent validation analyses together with their corresponding miRNAs are highlighted with black circle/diamond borders.

*In silico* analysis of potential microRNAs regulating the meta-signature genes predicted interactions between 30 meta-signature genes and 348 microRNAs. Using the list of the predicted interactions, we investigated if these potentially interacting microRNAs are significantly regulated in our endometrial microRNA-sequencing data on endometrial biopsies from the mid-secretory phase *vs*. early secretory phase of healthy fertile women. We identified 19 microRNAs that were significantly down-regulated in the mid-secretory endometria with corresponding 11 meta-signature genes to be significantly up-regulated in our sample set (Fig. 6). Based on the TargetScan context++ scores, the probability of the interaction between microRNA and its target gene seems to be higher in pairs miR-449c-5p and *DKK1*, miR-450b-5p, miR-424-5p, miR-130b-3p and *IL15*, miR-500a-5p and *GADD45A*, and miR-181a-2-3p and *ACADSB*. When focussing only on the genes that were confirmed in both independent validation analyses (RNA-seq of endometrial biopsies and cell type-specific RNA-seq), five target genes (*ANXA4*, *ARID5B*, *DKK1*, *EFNA1* and *SPP1*) and 10 microRNAs remained important (Fig. 6).

## Discussion

In this report, we present a systematic review and meta-analysis approach together with comprehensive experimental validation in order to identify promising biomarkers and molecular pathways involved in mid-secretory endometrial functions. Analysing the lists of differentially expressed genes from previously published expression profiling studies, we established a meta-signature of receptive endometrium with 57 genes as putative receptivity biomarkers. Interestingly, the commercial transcriptome-based endometrial receptivity diagnostic tool ERA (Endometrial Receptivity Array)^7, 30^ shares 47 genes in common with the identified meta-signature. Validation of the meta-signature genes in two different sample sets of healthy fertile women in mid-secretory *vs*. early secretory endometria using the up-to-date transcriptome analysis by RNA-seq confirmed 39 meta-signature genes.

The human endometrial transcriptome has been extensively studied in the past decade in a search of identifying diagnostic markers of receptive endometrium and to provide more understanding into the complex regulation of endometrial functions. Despite of the mass ‘omics’ data generated, only three *in silico* data-mining studies^17, 31, 32^ using previously published gene expression data have been published to date. Bhagwat *et al*. created a Human Gene Expression Endometrial Receptivity database (HGEx-ERdb) of 19,285 genes expressed in human endometrium, among which they identified 179 receptivity-associated genes^32^. Zhang *et al*. analysed raw data from three previous microarray studies^33–35^ and proposed 148 potential biomarkers of receptive endometrium^17^, while Tapia *et al*. integrated gene lists from seven previous microarray studies and presented a list of 61 endometrial receptivity biomarkers^31^. These three *in silico* analysis studies share only nine genes in common, highlighting the differences not only in *in silico* analysis approaches applied but also the great variation in study designs, analysis methods and data processing in published transcriptome studies. Clearly the mass of data generated within endometrial transcriptomics studies is under-explored, challenging investigators in future to analyse huge sets of data simultaneously in order to raise power, credibility and reliability of the findings.

The preferred method for gene expression meta-analysis requires analysis of raw expression datasets. However, such a thorough analysis is often not possible as a result of unavailability of raw data, which is partially the case in our meta-analysis. Variation in the number of gene transcripts known at a given moment together with the technological platform employed makes proper integration of raw datasets complicated. In addition, the limited sample size and noisiness of microarray data have resulted in inconsistency of biological conclusions^36^. In order to overcome these limitations, we directly analysed lists of differentially expressed genes from nine published studies involving a total of 164 endometrial biopsy samples from healthy women. Using a method that has been specifically designed for comparison of gene lists and identification of commonly overlapping genes in various studies, including recently published transcriptome studies in different ethnic groups^30, 37, 38^, we hope to provide an up-to-date meta-signature of endometrial receptivity biomarkers. Nevertheless, we have to bear in mind that with our approach, analysing the significantly differentially expressed gene lists, we could have missed the potential biomarker genes that were below statistical significance in individual studies but could become relevant in a meta-analysis.

The 57 genes identified in our meta-analysis could serve as the top-priority biomarkers of receptive phase endometrium in humans. Of special interest is *SPP1*, which was detected in all transcriptome studies that were included in our meta-analysis, together with *ANXA4*, *CLDN4*, *DPP4*, *GPX3*, *MAOA*, and *PAEP*, as they have also been identified as putative biomarkers of endometrial receptivity in the previous data-mining and review studies^17, 19, 29, 31, 32^.

Secreted phosphoprotein 1, SPP1, also known as osteopontin, is a secreted extracellular matrix (ECM) protein that binds to different cell-surface integrins to stimulate cell–cell and cell–ECM adhesion and communication (see Fig. 2), which play a part in the implantation process in various species^39–41^. It is generally accepted that SPP1 interacts with apically expressed integrins on the luminal endometrial epithelium and embryo trophectoderm to attach the conceptus to the endometrium^39^. Indeed, our cell type-specific RNA-seq validation analysis of endometrial epithelial and stromal cells demonstrates that *SPP1* is up-regulated only in the epithelial cells (though in our setting we had a mixture of both luminal and glandular epithelial cells) and not in the stromal cells in the receptive phase endometrium (Fig. 5). Dysregulation of osteopontin in mid-secretory endometria of women with various reproductive disorders has been detected in several studies^42–46^. Further, our previous systems biology approach in investigation of the molecular networks in the implantation process revealed the involvement of osteopontin together with leukemia inhibitory factor (LIF), apolipoprotein D (APOD) and leptin (LEP) pathways intertwining in a large network of cytokine–cytokine receptor interactions^37^.

Our meta-signature of mid-secretory endometrium highlights the importance of defence responses, specifically the inflammatory response (now recognized as a type of non-specific immune response), immunoglobulin-mediated immune responses (humoral immunity) and the complement (major mediator of innate immunity), and coagulation cascade pathway in receptive-phase endometrium. Immune responses, including the inflammatory response, play important roles in the pre- and peri-implantation period, and the up-regulation of genes involved in immune responses during the mid-secretory phase was corroborated in our meta-analysis and has also been highlighted in several previous studies^20, 31, 47–49^. In order to provide a hospitable environment for the embryo, the balance should be established between the maternal immune tolerance toward a semi-allogeneic implanting embryo and the protective anti-infectious mechanisms in the receptive-phase uterus^47, 49^. The innate immune system is the first line of defence, providing an immediate response through its ability to distinguish between ‘infectious non-self’ and ‘non-infectious self’ antigens^50^. Our meta-analysis highlights the importance of five genes involved in innate immunity, specifically in the complement system in mid-secretory endometrium, i.e. *C1R*, *SERPING1*, *CD55*, *C4BPA* and *CFD*, as shown in Fig. 2. CD55 (also known as DAF), for instance, is a complement regulatory protein with two suggested functions: protection of the embryo from maternal complement-mediated attack, and prevention of epithelial destruction resulting from increased complement expression at the time of implantation^51^. This protein has been found to be expressed at decreased levels in the endometria of women with recurrent pregnancy loss with antiphospholipid syndrome^52^. C4BPA is also suggested to have an embryo-protective role, where increased expression of this inhibitor of complement system activation could reduce the possibility of an uncontrolled complement attack on embryo^31^. Abnormally decreased levels of *C4BPA* expression in mid-secretory phase endometrium have been detected among women with endometriosis^53, 54^, implantation failure^55^ and unexplained recurrent abortion^56^.

The finding that a significant proportion of meta-signature genes are located in extracellular regions, including extracellular vesicles/exosomes, is intriguing. It is well known that the luminal epithelium with its extracellular area is the first maternal surface to interact with the trophoblast cells of the implanting embryo, but the involvement of extracellular vesicles in the implantation process is a new phenomenon^57–61^. Extracellular vesicles are membrane-bound complexes secreted from cells that act as messengers for cell–cell communication and signalling^62^. The origin of microvesicles and exosomes from endometrial epithelial cells in mid-secretory endometrium, the involvement of endometrial receptivity genes/proteins in exosomes and the uptake of extracellular vesicles by target blastocyst cells is depicted on Fig. 3. It has been proposed that extracellular vesicles, containing specific RNAs, including microRNAs and proteins, are released into the uterine cavity that could be transferred to either trophoblast cells or to endometrial epithelial cells, where they promote implantation^57, 58, 62, 63^. Twenty eight proteins from our endometrial receptivity-associated gene list have been experimentally detected in exosomes in humans (ExoCarta database). Our findings support the role of exosomes in endometrial receptivity and the subsequent embryo implantation, and indicate that further research into functional effects of extracellular vesicles in embryo-endometrium cross-talk is needed. Research on extracellular vesicles is a rapidly evolving and expanding field that could offer new opportunities regarding biomarkers of receptive endometrium and embryo implantation. Especially intriguing is the fact that extracellular vesicles have the potential in the development of non-invasive biomarkers and for thriving novel therapies to increase reproductive success.

The involvement of microRNAs in the mid-secretory endometrial functions has been shown by previous studies^18^. Further, studies on mice demonstrate that microRNAs are important in implantation and pregnancy, and the loss of Dicer (RNAse III endonuclease that is essential for the biogenesis of microRNAs) within uterus can compromise fertility^64^. MicroRNAs are non-coding RNA molecules acting as posttranscriptional regulators of gene expression and operate by either degrading or translationally repressing the target mRNAs^65^. There are now known over 2,000 annotated microRNAs in the human genome^66^, and since each microRNA may regulate hundreds of genes, it is estimated that microRNAs collectively regulate one third of genes in the genome^67^. Our prediction and subsequent validation analyses identified 19 down-regulated microRNAs in the mid-secretory phase endometria that resulted in up-regulation of 11 target mRNAs, because of reduced miRNA-mediated repression. Of special interest are miR-130b-3p and *ANXA4*, miR-548n and *SPP1*, miR-548ah-3p, miR-30c-1-3p and *EFNA1*, miR-30c-1-3p and *ARID5B*, and miR-449c-5p and *DKK1* pairs, where the meta-signature gene was validated in two independent validation analyses. The importance of miR-30 family members, miR-30b and miR-30d, in endometrial receptivity have been highlighted in different studies^58, 68–70^, however the changed expression of miR-30c-1 has been detected so far in endometrial cancer patients^71^. In porcine endometrium the expression of miR-30c has been shown to increase during the gestational days, meaning that at the time of implantation this microRNA has been down-regulated when compared to placentation and mid-gestational times^72^. The increased expression of miR-130b and miR-449c-5p have been detected in endometrial cancer patients when compared to controls^73, 74^.

With our meta-analysis we highlight highly potential biomarkers of endometrial receptivity, but their molecular mechanisms in uterine physiology and pathophysiology remain to be investigated. Furthermore, to our knowledge, none of the molecular markers have yet been successfully applied in clinical therapeutic practice, including the highly promising molecule LIF^15^. Hence, the hunt for potentially informative and therapeutic markers of uterine receptivity continues. The era of looking for endometrial receptivity markers at other ‘omics’ levels has begun and it is to be hoped that this will result in further promising results (reviewed by refs 18 and 75). We believe that a novel approach for the future could hold in the microRNAs and/or exosome-based testing and therapeutic strategy for improving endometrial receptivity. Regardless of the biomarker sets chosen to identify receptive endometrium, all will need extensive validation before their clinical utility can be proven. Several of our meta-signature genes have already been validated on mRNA and/or protein level in individual marker and/or transcriptome studies (summarised in Table 2).

In conclusion, we present a meta-analysis approach allowing convergence and dissection of heterogeneous mRNA expression profiling datasets of receptive phase endometrium. We identified a meta-signature of endometrial receptivity composed of 57 genes, where 39 of these genes were experimentally confirmed in two separate datasets. These meta-signature genes highlight the importance of immune responses, the complement cascade pathway and the involvement of exosomes in mid-secretory endometrial functions, and could serve as promising biomarkers of endometrial receptivity and achieving a pregnancy.

## Methods

### Systematic search of the literature

A systematic review of the literature in PubMed and Scopus was independently conducted up to June 2016 by two researchers (S.A. and M.S.). MeSH terms ‘embryo implantation’, ‘endometrium’ and ‘gene expression’ were used. The reference lists of review articles and relevant studies were hand-searched to identify other potentially eligible studies. No language or any other restrictions were applied. Details of the protocol for this systematic review were registered on PROSPERO and can be accessed at: http://www.crd.york.ac.uk/PROSPERO/display_record.asp?ID = CRD42016041509.

Abstracts of all articles identified through the search were read for selection of eligible studies. The full text of each eligible article was carefully evaluated. Only original experimental studies published in English and concerning the endometrial transcriptome (using microarray or RNA-seq techniques) in healthy women in the mid-secretory phase *vs*. the ‘pre-receptive’ phase (proliferative and/or early secretory phase) were included for final analysis. Endometrial transcriptome analysis in connection with any pathological condition, such as infertility, endometriosis, adenomyosis and cancer was excluded. In addition, transcriptome analyses focussing on different endometrial tissue-compartments separately were not included in the meta-analysis.

### Analysis settings

The lists of genes differentially expressed in mid-secretory *vs*. ‘pre-receptive’-phase endometrium were extracted from the publications. Where the gene lists were not available, the authors were contacted directly. In transcriptome array studies, if the probe information for a dataset was available, probes were annotated to corresponding ENTREZ IDs using respective Bioconductor 3.1 Affymetrix probe annotation package, in studies using Affymetrix microarray platform. If probe-level data was not available, gene lists were converted to ENTREZ IDs by using the DAVID Gene ID Conversion Tool (DAVID Bioinformatics Resources). Genes reported in a study by Hu *et al*. that involved Illumina RNA-seq platform^38^ were standardised to ENTREZ IDs by using the BiomaRt package and ENSEMBL v.75 database. For one study^33^, the differentially expressed probe lists were acquired by reanalysing the data stored in the Gene Expression Omnibus under accession number GSE6364. We used GEO2R web tool (http://www.ncbi.nlm.nih.gov/geo/info/geo2r.html) with default options for differential analysis and gene list acquisition (false discovery rate, FDR < 0.05; fold change, FC > 2.0). Probes not annotated by ENTREZ IDs were removed from subsequent analyses.

Subsequently, lists of up- and down-regulated genes were ranked by their fold changes. In cases of multiple probes detecting the same gene, only the probe with the largest absolute fold change was used for list construction.

### Meta-analysis

The robust rank aggregation algorithm (RRA package v.1.1) was used for meta-analysis of the ranked gene lists^24^. To assess full gene list sizes, the number of detectable gene ENTREZ IDs was used for each array platform and all ENTREZ IDs from BioMart v.75 were used for the RNA-seq study^38^. For correcting for multiple testing, we used a strict Bonferroni threshold by multiplying all P-values by the maximal number of elements in all input lists. In our case this involved the data published by Hu *et al*.^38^, where the total number of ENTREZ IDs available in ENSEMBL v.75 (39,030) was used as the total number of tests. All lists used in the analysis reflect expression in the mid-secretory group compared with another group (proliferative or early-secretory phase).

### Enrichment analysis

Enrichment analyses for Gene Ontology (GO) terms and biological pathways (KEGG and Reactome) were carried out by using the g:Profiler web tool (biit.cs.ut.ee/gprofiler/)^76, 77^. This software was chosen over other enrichment analysis tools as it is up-to-date (updated in May 2016 to Ensembl 84 and Ensembl Genomes 31) and it provides a compact graphical output. The obtained results were corrected for multiple testing by using the g:Profiler tailor-made algorithm g:SCS, which has been shown to provide a better threshold between significant and non-significant results than (commonly used) Bonferroni correction or the Benjamini-Hochberg false discovery rate^76^.

### *In silico* analysis of potential microRNAs regulating meta-signature genes

MicroRNA target prediction was performed using three different algorithms – DIANA microT-CDS, TargetScan 7.0 and miRanda v3.3a. In DIANA microT-CDS^78^ and TargetScan 7.0^79^ precomputed prediction results were downloaded from their respective websites (diana.imis.athena-innovation.gr/DianaTools/index.php?r = microT_CDS/index; www.targetscan.org/cgi-bin/targetscan/data_download.cgi?db = vert_70). DIANA microT-CDS utilises ENSEMBL v69 transcriptome and miRBase v18 for the prediction, whereas TargetScan 7.0 uses ENSEMBL v75 and miRBase v21^80^. miRanda v3.3a binary^81^ was downloaded from http://www.microrna.org/microrna/getDownloads.do and used for the target prediction with ENSEMBL v75 3′UTRs and miRBase v21 mature sequences as an input. The algorithm was used with default settings: Gap Open Penalty: −9.0, Gap Extend Penalty: −4.0, Score Threshold: 140.0, Energy Threshold: 1.0 kcal/mol and Scaling Parameter: 4.0. miRBase internal IDs were used to standardise microRNA names between different miRBase versions.

For additional support to *in silico* target predictions, we used database harbouring experimental data about mammalian microRNA binding sites. Therefore, Argonaute (AGO1, AGO2, AGO3 and AGO4) HITS-CLIP and PAR-CLIP datasets for human cell lines were downloaded from StarBase v.2.0^82^ website in the BED format and overlaid with predicted microRNA target sites (http://starbase.sysu.edu.cn/download.php).

### Validation of meta-signature genes and predicted microRNAs in the independent sample sets

We validated the meta-signature genes in our two independent sample sets from NOTED project (EU-FP7 Eurostars Programme, EU41564) and SARM project (EU-FP7, IAPP, EU324509). The studies were approved by the local Research Ethics Committees of the University of Tartu and Instituto Valenciano de Infertilidad. An informed consent was signed by all women who agreed to participate in the study, and all methods were carried out in accordance with relevant guidelines and regulations.

The detailed description of the study participants and RNA-seq analysis within NOTED project is described in the Supplementary Material. Briefly, 20 healthy fertile women provided endometrial biopsy samples in the early secretory phase (2 days after the luteinizing hormone (LH) peak, LH+2) and in the mid-secretory phase of the menstrual cycle (LH+8) within the same natural cycle. Total mRNA transcriptome and microRNA profile analysis from the same biopsy samples were performed with the RNA-seq method. Differential expression was tested using the edgeR statistical package. Up-regulation was defined as statistically significantly (FDR corrected p-value < 0.05) higher expression (expressed as ‘counts per million reads’, CPM) in the mid-secretory phase samples, whereas down-regulation was defined as statistically significantly lower expression in the mid-secretory samples. The primary RNA-seq data are available in the public database Gene Expression Omnibus (www.ncbi.nlm.nih.gov/geo/) under accession number GSE98386.

The other independent set of validation was carried out in additional 16 healthy fertile women within SARM project, where cell type-specific RNA-seq on endometrial samples was performed, with separated epithelial and stromal cells. Briefly, endometrial biopsies were collected from 16 healthy fertile women on two different time points within the natural cycle, LH+2 and LH+8. Single cells from endometrial biopsy samples were separated, epithelial cells were labelled with fluorochrome-conjugated mouse anti-human CD9 monoclonal antibody and stromal cells were simultaneously labelled with fluorochrome-conjugated mouse anti-human CD13 monoclonal antibody, followed by flow cytometric analysis and FACS cell sorting. Bulk-RNA full transcriptome analysis of FACS sorted endometrial epithelial and stromal cells was performed with the RNA-seq method, following the single-cell tagged reverse transcription (STRT) protocol with modifications^83^. Differential expression was tested using the edgeR statistical package. Up-regulation was defined as statistically significantly (FDR corrected p-value < 0.05) higher expression (expressed as ‘normalised read counts’) in the mid-secretory phase samples, whereas down-regulation was defined as statistically significantly lower expression in the mid-secretory samples. The primary RNA-seq data are available in the public database Gene Expression Omnibus (www.ncbi.nlm.nih.gov/geo/) under accession number GSE97929. Detailed protocol of the sample collection, processing, and analysis is described in the Supplementary Material.

Further validation of the endometrial receptivity signature genes *DDX52*, *DYNLT3*, *C1R* and *APOD* on NOTED and SARM project samples using real-time PCR is described in the Supplementary Material.

## Electronic supplementary material

Supplementary Material

Supplementarty Table 2

Supplementary Table 3
