# Supplementary material for: Meta-signature of human endometrial receptivity: a meta-analysis and validation study of transcriptomic biomarkers
Source: Sci Rep. 2017 Aug 30;7:10077. doi: 10.1038/s41598-017-10098-3 (PMC5577343; doi:10.1038/s41598-017-10098-3)
Supplement: Supplementary file 1 — Supplementary Material [file 41598_2017_10098_MOESM1_ESM.pdf]

Meta-signature of human endometrial receptivity: a meta-analysis and validation study of transcriptomic biomarkers

Signe Altmäe, Mariann Koel, Urmo Võsa, Priit Adler, Marina Suhorutšenko, Triin Laisk-Podar, Viktorija Kukushkina, Merli Saare, Agne Velthut-Meikas, Kaarel Krjutškov, Lusine Aghajanova, Parameswaran G. Lalitkumar, Kristina Gemzell-Danielsson, Linda Giudice, Carlos Simón, Andres Salumets

Supplementary Material

Validation of meta-signature genes and their regulatory microRNAs on independent sample set from NOTED project

Study participants

The study was approved by the local Research Ethics Committee of the University of Tartu and an informed consent was signed by all women who agreed to participate in the study. Endometrial biopsies were obtained from 20 healthy volunteers at fertile age (≤ 35 years) with normal BMI (within a range of 19-25). All women selected for the study had regular menstruation and were clinically examined for the absence of hormonal aberrations and had no uterine pathologies, endometriosis or polycystic ovary syndrome. The women were non-smokers, were not taking any hormonal treatments for three months prior to the study, had no previous infertility record, and had at least one live-born child.

The biopsy was obtained using Pipelle catheter (Laboratoire CCD, Paris, France) on day 2 and 8 after the luteinizing hormone (LH) surge (LH+2 and LH+8) within the same natural cycle. Menstrual cycle dating was confirmed by combining LH testing (estimated by the BabyTime hLH urine cassette, Pharmanova), vaginal

ultrasound and histological evaluation of biopsy according to the Noyes' criteria [1] in order to confirm the receptive phase of endometrial maturation. The endometrial tissue was frozen after biopsy at -80°C in RNAlater (Ambion Inc., Austin, TX) for further analysis.

Total RNA and microRNA analyses

Total RNA was extracted from up to 30mg of endometrial tissue. The biopsy was homogenized in the presence of QIAzol reagent (Qiagen, Venlo, Netherlands) and processed using miRNeasy Mini kit following the manufacturer's protocol (Qiagen). microRNA content was enriched from total RNA using Qiagen's MinElute protocol. Purified RNA quality was determined with Bioanalyzer (Agilent Technologies, Waldbronn, Germany) and RNA integrity value (RIN) >7 was considered acceptable. In order to perform transcriptome sequencing, cDNA libraries were generated from ~1μg of endometrial total RNA using Illumina TruSeq kit (Illumina, San Diego, CA, USA), following cDNA quality control with Bioanalyzer. For microRNAs, libraries were generated using TruSeq Small RNA kit. The outcome of paired-end 100 bp sequencing on Illumina HiSeq2500 instrument was subjected to bioinformatical analysis.

RNA-seq data processing

The RNA raw read quality was controlled with FastQC v.0.11.3 before and after preprocessing steps. The preprocessing included the adapter removal and trimming with Trimmomatic tool v.0.32 and low quality read filtering with fastq_quality_filter from fastx toolkit v.0.0.13. High quality reads were aligned to the human reference genome hg19 using TopHat v.2.0.11 aligning tool. The downstream analysis was based on the clean high quality data. The read counts were counted with the HTSeq package script

htseq-count v.0.6.1, using the gtf Ensembl v.72 annotation file as the genomic feature file and aligned reads as the input. The Bioconductor package edgeR v.3.6.2 was used for the paired differential analysis and as the package requests, the raw read counts were used as an input. For the analysis, transcripts with CPM (counts per million) >2 at least in 15 of 40 samples were used. The multiple testing P-values were adjusted using the Benjamini-Hochberg's approach to control the false discovery rate (FDR). All the transcripts with FDR <0.05 were identified as differentially expressed.

Raw reads from small RNA sequencing were pre-processed with Trimmomatic tool and fastq_quality_filter. High quality reads were mapped and raw read counts quantified with miRDeep2 package (12.08.2009). For microRNA mapping we used human reference genome hg19, mature microRNA and hsa-loops annotation files from miRBase v. 21 (June 2014). The paired differential analysis for microRNA data was performed with edgeR using raw counts. The microRNAs with CPM >1 in at least 15 out of 40 samples were used for analysis. The microRNAs with FDR <0.05 were identified as differentially expressed.

Validation of meta-signature genes on independent sample set from SARM project

Study participants

The study was approved by the local Research Ethics Committees of the University of Tartu and Instituto Valenciano de Infertilidad, and an informed consent was signed by all women who agreed to participate in the study.

Endometrial biopsies were collected from 16 healthy fertile women from Estonia and Spain using an endometrial suction Pipelle catheter (Laboratoire CCD, Paris, France). Two samples were obtained from every woman, one on the early-secretory phase (2 days after the LH peak, LH+2) and second from the mid-secretory

phase of the menstrual cycle, LH+8. All women selected for the study were at fertile age (≤ 35 years), had normal BMI (19-25), reported regular menstruation and were clinically examined for the absence of hormonal aberrations and had no uterine pathologies, endometriosis or polycystic ovary syndrome. All women were non-smokers, not having any hormonal treatment, had no previous infertility record, and had at least one live-born child.

FACS sorting of single endometrial cells

Handling, dissociation and preparation of the biopsies for the FACS sorting is described in detail in our recent publication [2]. Briefly the endometrial tissue samples were placed immediately into the cryopreservation medium and were frozen in a Nalgene® Cryo 1°C 'Mr. Frosty' Freezing Container (Thermo Fisher Scientific, Waltham, MA, USA) in −80°C freezer overnight. The usage of proper media and moderate freezing preserves intact cells, and provides living cells for further specific antibody labelling and sorting steps.

The biopsy was dissociated in 5 ml DMEM medium containing 0.5% collagenase (Sigma-Aldrich, St. Louis, USA) in a shaking incubator rotating at 110 rpm at 37°C until the biopsy was digested in <20 min. 500 μl of ice-cold FBS and 45 ml of ACK lysing buffer (Life Technologies, Carlsbad, CS, USA) were added and the suspension was centrifuged at 205 × g 4°C for 6 min. The cells were re-suspended in 4 ml ice-cold phosphate buffered saline (PBS) containing 5% fetal bovine serum (FBS) solution and the suspension was filtered twice through 50 and 35μm Falcon Tube with Cell Strainer Cap (BD Biosciences, San Jose, CA, USA) to separate single cells from undigested endometrial tissue fragments. The filtrate was centrifuged at 210 × g 4°C for 6 min to collect cells and re-suspended in 200 μl of PBS/FBS solution. Endometrial

stromal cells were stained with fluorochrome-conjugated mouse anti-human CD13 monoclonal antibody (1:20 dilution, clone TÜK1, R-Phycoerythrin) (Thermo Fisher Scientific) and epithelial cells were stained simultaneously with fluorochrome-conjugated mouse anti-human CD9 monoclonal antibody (1:5 dilution, clone MEM-61, FITC) (Novus Biologicals, Littleton, CO, USA) on ice for at least 15 min. After incubation 1.8 ml ice-cold PBS/FBS solution was added and the mixture was centrifuged at 210 × g 4°C for 5 min. The cells were suspended in 300 µl PBS/FBS solution and filtered using 35 µm Falcon Tube with Cell Strainer Cap (Thermo Fisher Scientific). Filtered cells were stained with DAPI (1 mg/ml, 1:2000 dilution) (Thermo Fisher Scientific) to exclude dead cells. Cell suspensions were maintained at 4°C until flow cytometric analysis and cell sorting. CD9 or CD13 positive and DAPI negative (alive) cells were sorted into a QIAzol Lysis Reagent and RNAs were extracted immediately after the lysis of cells.

Cell type-specific RNA analysis and RNA-seq data processing

Bulk-RNA full transcriptome analysis of FACS sorted endometrial cells was performed with the RNA-seq method, following the single-cell tagged reverse transcription (STRT) protocol with modifications [2]. Instead the RNA from single cell, 10ng of high-quality input RNA was converted to cDNA and amplified to form an Illumina-compatible library. The STRTprep pipeline v.3.0.0, available at https://github.com/shka/STRTprep, was used for processing raw sequencing reads, aligning to the hg19 genome. Spike-in-based normalisation and differential expression analyses were performed with the R package edgeR (v.3.12.0) [3], with FDR of <0.05 used as threshold for differential expression. All the pipeline steps are usable in the same manner for the bulk RNA as for single-cell RNA analysis with minor

modifications. The gene expression was compared between early- (LH+2) and mid-secretory (LH+8) phase for CD9-positive epithelial cells and CD13-positive stromal cells.

Validation of RNA-seq analyses using quantitative real-time PCR

C1R, *APOD*, *DYNLT3* and *DDX52* expression levels were determined in 10 paired LH+2 and LH+8 endometrial samples and five paired LH+2 and LH+8 endometrial FACS-sorted endometrial epithelial and stromal cells. DNase treated (TURBO DNA-free™ kit, Ambion Inc., Austin, Texas, USA) RNA was converted into cDNA using RevertAid First Strand cDNA Synthesis Kit (Thermo-Fisher Scientific Inc. MA, USA). qRT-PCR was performed using 1 × HOT FIREPol EvaGreen qPCR Mix Plus (Solis BioDyne, Estonia) according to the conditions specified by the manufacturer. The following primers were used: *C1R* (Rev-GACCCTAGACGAGTTCACCA, Fw-CCACGTGCCATCATCCTG); *DYNLT3* (Rev-GCGACGAGGTTGGCTTCAA, Fw-GCTTTTCCCAACTTAACCAGGT); *APOD* (Rev-TGAATCAAATCGAAGGTGAAG, Fw-GTGCCGATGGCATAAACC) and *DDX52* (Rev-TCCATTGAAAGGGCTAAAGAACT, Fw-GGACTGTGTTATCTCTCTGTTGT). The *SDHA* (Rev-CCACCACTGCATCAAATTCATG, Fw-TGGGAACAAGAGGGCATCTG) was used as endogenous control. The expression differences between LH+2 and LH+8 were calculated using Student's t-test, using a p-value cut-off of $p < 0.05$. The $2^{-\Delta\Delta Ct}$ method [4] was used for calculating the relative expression between LH+2 and LH+8 samples.

References

1. Noyes, R. W., Hertig, A. T. & Rock, J. Dating the endometrial biopsy. *Am J Obs. Gynecol* **122,** 262–263 (1975).

2. Krjutškov, K. *et al.* Single-cell transcriptome analysis of endometrial tissue. *Hum. Reprod.* **31,** 844–53 (2016).
3. Robinson, M. D. & Oshlack, A. A scaling normalization method for differential expression analysis of RNA-seq data. *Genome Biol.* **11,** R25 (2010).
4. Livak, K. J. & Schmittgen, T. D. Analysis of relative gene expression data using real-time quantitative PCR and the 2(-Delta Delta C(T)) Method. *Methods* **25,** 402–8 (2001).

Supplementary Information

Supplementary Figure 1. Prisma Flow Diagram: systematic review process of the literature from initial search to final inclusion of studies in the meta-analysis.

Supplementary Figure 2. FACS-sorted cell type-specific RNA-seq results of the 57 meta-signature genes in independent sample set. Y-axis denotes the $\log_{10}$ normalised read count values and the x-axis indicates the endometrial cycle phase (E_2 – epithelial cells at LH+2, E_8 – epithelial cells at LH+8, S_2 – stromal cells at LH+2, S_8 – stromal cells at LH+8). FDR values of significantly regulated genes are shown.

Supplementary Figure 3. Real-time PCR validation of *DDX52*, *DYNLT3*, *C1R* and *APOD* genes. Y-axis denotes the relative gene expression levels (negative value of relative gene expression (-1x(Ct(gene of interest)-Ct(control gene))). Up-regulated gene expression level was confirmed in: 1) endometrial whole tissue samples in the mid-secretory *vs.* early secretory phase for all analysed genes; 2) FACS-sorted epithelial cells in the mid-secretory *vs.* early secretory phase for *DDX52* and *DYNLT3* genes; and 3) FACS-sorted stromal cells in the mid-secretory *vs.* early secretory phase for *C1R* and *APOD* genes. Respective p-values of mid-secretory (LH+8) *vs.* early secretory (LH+2) phase comparisons are indicated.

Supplementary Table 1. Gene ontology (GO) processes and the pathways most strongly enriched among endometrial receptivity-associated genes.

Supplementary Table 2. *In silico* analysis of potential microRNAs regulating meta-signature genes. Three different prediction algorithms – DIANA microT-CDS, TargetScan 7.0 and miRanda v3.3a were used. Scores for each analysis are indicated. The overlap between all three algorithms resulted in 818 microRNAs and 1,403 potential unique binding sites for 43 meta-signature genes. The table includes also microRNA binding sites that differ in 1-2 nucleotides, hence in total 1,909 binding sites for 43 genes are presented.

Supplementary Table 3. The consensus list of predicted microRNAs and their target genes based on the different prediction algorithms applied (DIANA microT-CDS, TargetScan 7.0 and miRanda v3.3a) and AGO-CLIP dataset of experimentally determined Argonaute binding sites.

PRISMA 2009 Flow Diagram

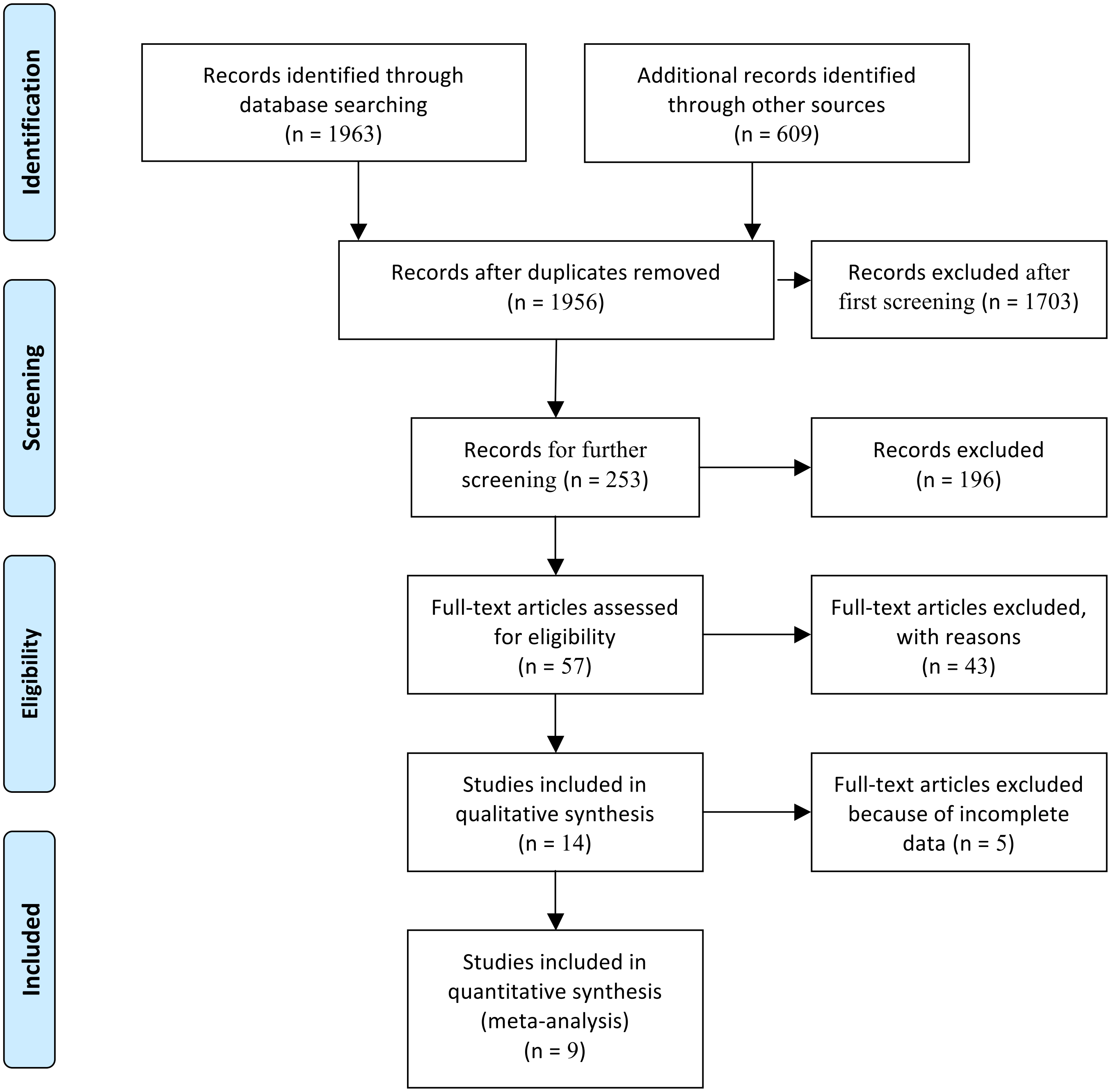

From: Moher D, Liberati A, Tetzlaff J, Altman DG, The PRISMA Group (2009). *P*referred *R*eporting *I*tems for *S*ystematic Reviews and *M*eta-*A*nalyses: The PRISMA Statement. PLoS Med 6(6): e1000097. doi:10.1371/journal.pmed1000097

For more information, visit www.prisma-statement.org.

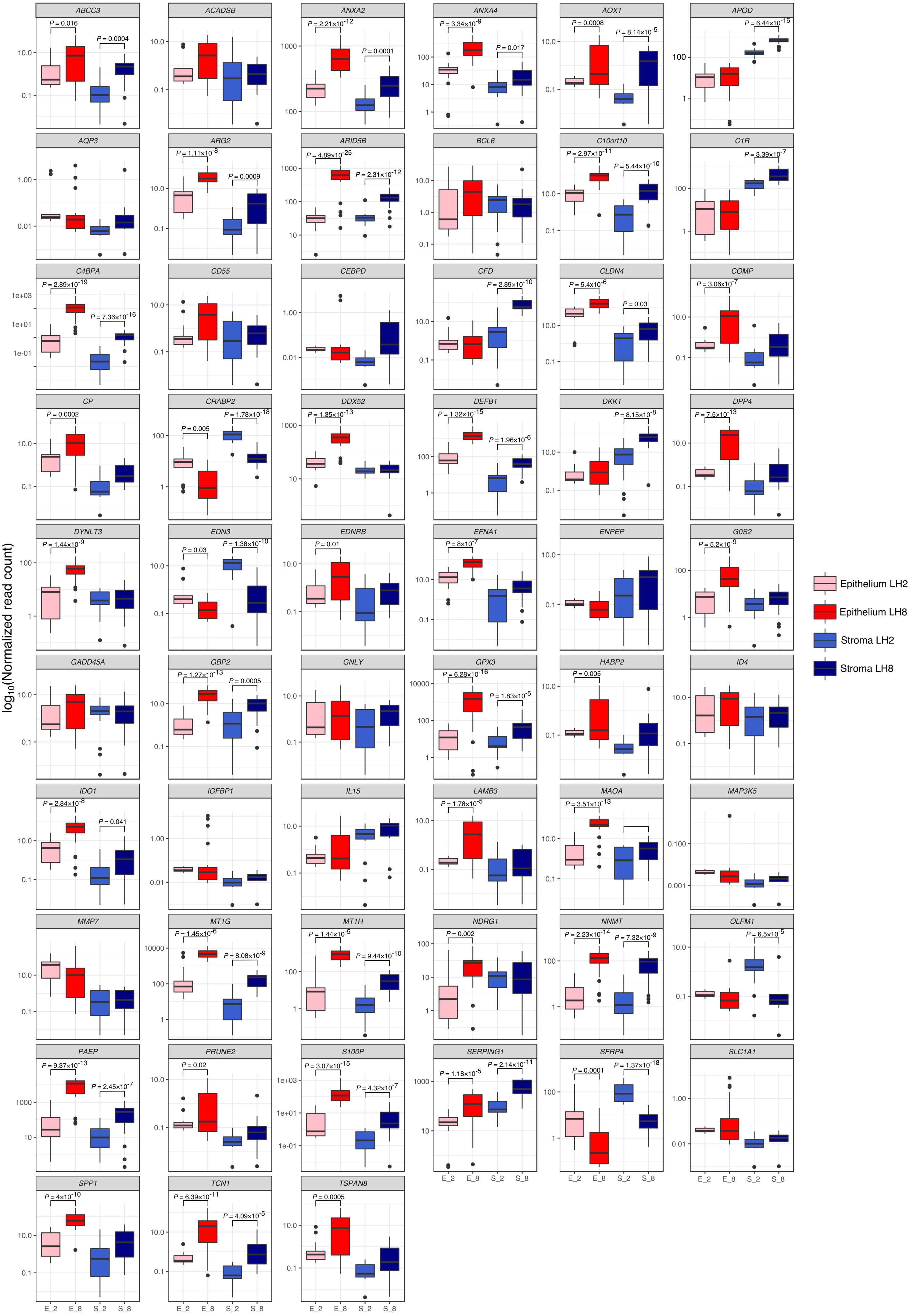
log$_{10}$(Normalized read count)
ABCC3 $P = 0.016$ $P = 0.0004$
ACADSB
ANXA2 $P = 2.21\times10^{-12}$ $P = 0.0001$
ANXA4 $P = 3.34\times10^{-9}$ $P = 0.017$
AOX1 $P = 0.0008$ $P = 8.14\times10^{-5}$
APOD $P = 6.44\times10^{-16}$
AQP3
ARG2 $P = 1.11\times10^{-8}$ $P = 0.0009$
ARID5B $P = 4.89\times10^{-25}$ $P = 2.31\times10^{-12}$
BCL6
C10orf10 $P = 2.97\times10^{-11}$ $P = 5.44\times10^{-10}$
C1R $P = 3.39\times10^{-7}$
C4BPA $P = 2.89\times10^{-19}$ $P = 7.36\times10^{-16}$
CD55
CEBPD
CFD $P = 2.89\times10^{-10}$
CLDN4 $P = 5.4\times10^{-6}$ $P = 0.03$
COMP $P = 3.06\times10^{-7}$
CP $P = 0.0002$
CRABP2 $P = 0.005$ $P = 1.78\times10^{-18}$
DDX52 $P = 1.35\times10^{-13}$
DEFB1 $P = 1.32\times10^{-15}$ $P = 1.96\times10^{-6}$
DKK1 $P = 8.15\times10^{-8}$
DPP4 $P = 7.5\times10^{-13}$
DYNLT3 $P = 1.44\times10^{-9}$
EDN3 $P = 0.03$ $P = 1.38\times10^{-10}$
EDNRB $P = 0.01$
EFNA1 $P = 8\times10^{-7}$
ENPEP
G0S2 $P = 5.2\times10^{-9}$
GADD45A
GBP2 $P = 1.27\times10^{-13}$ $P = 0.0005$
GNLY
GPX3 $P = 6.28\times10^{-16}$ $P = 1.83\times10^{-5}$
HABP2 $P = 0.005$
ID4
IDO1 $P = 2.84\times10^{-8}$ $P = 0.041$
IGFBP1
IL15
LAMB3 $P = 1.78\times10^{-5}$
MAOA $P = 3.51\times10^{-13}$
MAP3K5
MMP7
MT1G $P = 1.45\times10^{-6}$ $P = 8.08\times10^{-9}$
MT1H $P = 1.44\times10^{-5}$ $P = 9.44\times10^{-10}$
NDRG1 $P = 0.002$
NNMT $P = 2.23\times10^{-14}$ $P = 7.32\times10^{-9}$
OLFM1 $P = 6.5\times10^{-5}$
PAEP $P = 9.37\times10^{-13}$ $P = 2.45\times10^{-7}$
PRUNE2 $P = 0.02$
S100P $P = 3.07\times10^{-15}$ $P = 4.32\times10^{-7}$
SERPING1 $P = 1.18\times10^{-5}$ $P = 2.14\times10^{-11}$
SFRP4 $P = 0.0001$ $P = 1.37\times10^{-18}$
SLC1A1
SPP1 $P = 4\times10^{-10}$
TCN1 $P = 6.39\times10^{-11}$ $P = 4.09\times10^{-5}$
TSPAN8 $P = 0.0005$
E_2 E_8 S_2 S_8
Epithelium LH2
Epithelium LH8
Stroma LH2
Stroma LH8

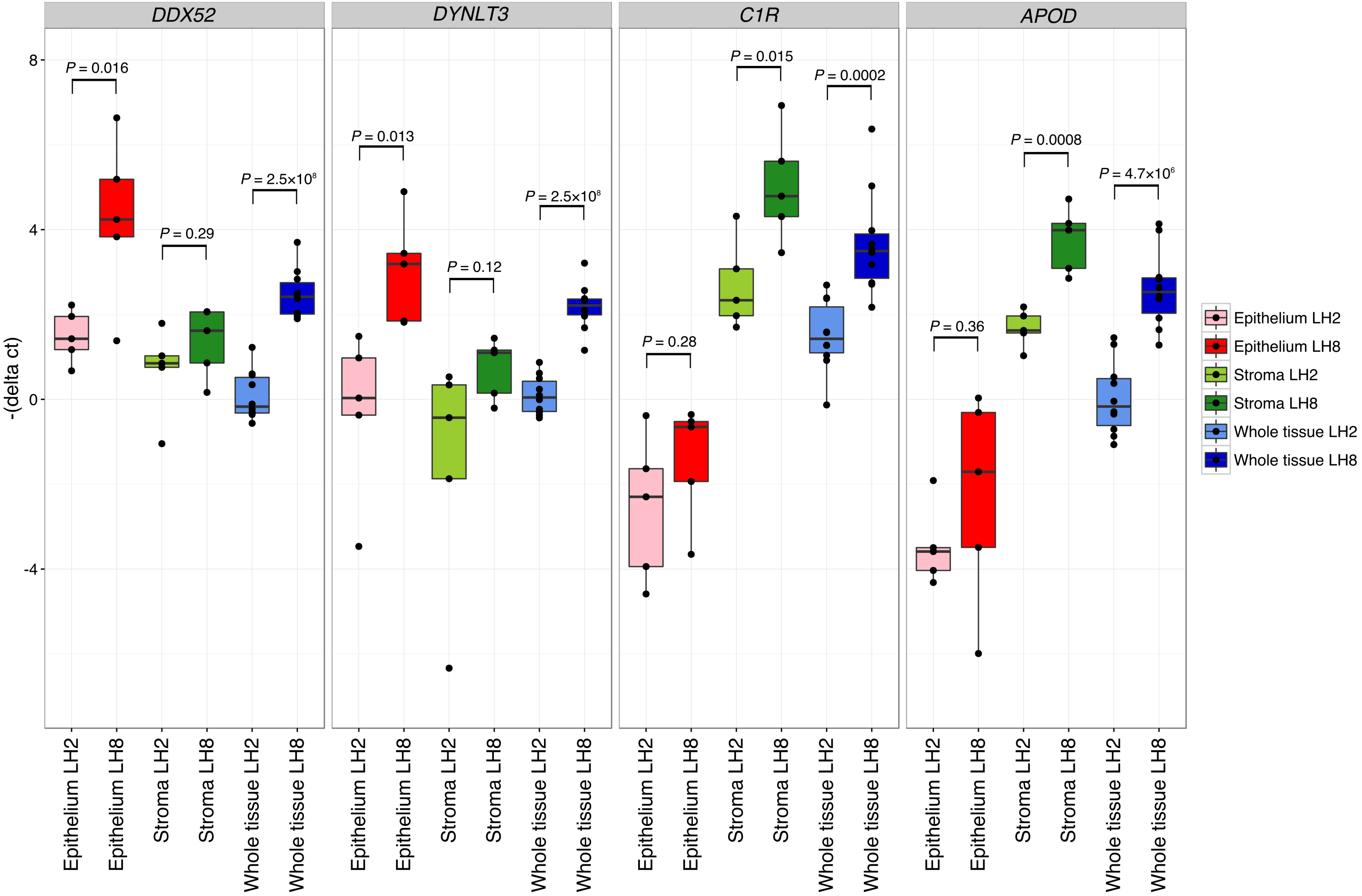
DDX52
DYNLT3
C1R
APOD
-(delta ct)
8
4
0
-4
P = 0.016
P = 0.29
$P = 2.5\times10^{8}$
P = 0.013
P = 0.12
$P = 2.5\times10^{8}$
P = 0.28
P = 0.015
P = 0.0002
P = 0.36
P = 0.0008
$P = 4.7\times10^{6}$
Epithelium LH2
Epithelium LH8
Stroma LH2
Stroma LH8
Whole tissue LH2
Whole tissue LH8

Supplementary Table 1. Gene ontology (GO) processes and the pathways most strongly enriched among endometrial receptivity-associated genes.

**GO term**	**Gene involved**
Response to external stimulus	*GADD45A, AQP3, BCL6, EDNRB, IDO1, IL15, GNLY, APOD, SERPING1, SPP1, DEFB1, C4BPA, CD55, EDN3, EFNA1, MMP7, TSPAN8, GBP2, ANXA2*
Negative regulation of coagulation	*SERPING1, TSPAN8, ANXA2, ANXA4*
Ig mediated immune response	*BCL6, SERPING1, C4BPA, CD55, C1R*
Inflammatory response	*BCL6, EDNRB, IDO1, IL15, APOD, SERPING1, SPP1, DEFB1, C4BPA, CD55, AOX1*
Humoral immune response	*SERPING1, DEFB1, C4BPA, CD55, MMP7, CFD, C1R*
Response to wounding	*BCL6, EDNRB, IDO1, IL15, APOD, SERPING1, SPP1, C4BPA, CD55, TSPAN8, ANXA2, CFD, IGFBP1*
Defence response	*BCL6, EDNRB, IDO1, IL15, GNLY, APOD, SERPING1, SPP1, DEFB1, C4BPA, CD55, MMP7, GBP2, AOX1, MAP3K5, CFD, C1R*
Negative regulation of multicellular organismal process	*BCL6, EDNRB, IDO1, APOD, SERPING1, SPP1, EFNA1, TSPAN8, ANXA2, ANXA4, OLFM1, DKK1, ID4*
Protein activation cascade	*SERPING1, C4BPA, CD55, CFD, C1R*
Immune system process	*AQP3, BCL6, EDNRB, IDO1, IL15, APOD, SERPING1, SPP1, DEFB1, C4BPA, CD55, EDN3, MMP7, GBP2, ANXA2, MAP3K5, CFD, NDRG1, C1R, MT1G, DPP4*
Complement activation	*SERPING1, C4BPA, CD55, CFD, C1R*
Extracellular region, exosome	*IL15, GNLY, APOD, SERPING1, SPP1, DEFB1, C4BPA, CD55, EDN3, EFNA1, MMP7, TSPAN8, GBP2, ANXA2, AOX1, ANXA4, OLFM1, CFD, NDRG1, DKK1, C1R, DPP4, IGFBP1, COMP, CP, ACADSB, CRABP2, ENPEP, GPX3, HABP2, LAMB3, PAEP, S100P, SFRP4, SLC1A1, TCN1*
